# Supplementary material for: A cluster randomized controlled trial to assess the impact of the ‘Caring for Providers to Improve Patient Experience’ (CPIPE) intervention in Kenya and Ghana: study protocol
Source: BMC Public Health. 2024 Sep 16;24:2509. doi: 10.1186/s12889-024-20023-9 (PMC11403968; doi:10.1186/s12889-024-20023-9)
Supplement: Supplementary file 2 — Supplementary Material 2 [file 12889_2024_20023_MOESM2_ESM.doc]

**University of California, San Francisco (UCSF)**

**Kenya Medical Research Institute (KEMRI)**

**Navrongo Health Research Center (NHRC)**

| ***IRB NUMBERS*** | | |
| --- | --- | --- |
| ***UCSF-CHR:*** 23-38843 | ***SERU:*** | ***NHRC:*** |

***Title of Study:*** *Caring for Providers to Improve Patient Experience (CPIPE) Trial*

**Principal Researchers:**

| **Name** | **Role** | **Institution** |
| --- | --- | --- |
| Patience Afulani, MBChB, MPH, PhD | Principal Investigator | UCSF |
| Linnet Ongeri, MBChB, MMed, PhD | Site Principal Investigator- Kenya | KEMRI |
| Raymond Aborigo, PhD | Site Principal Investigator- Ghana | NHRC |

**24-hour Emergency Telephone Number: IN KENYA:** +254 790300073

**Introduction:** We are asking you to consider taking part in a research study done by Kenya Medical Research Institute (KEMRI), Navrongo Health Research Center (NHRC) and the University of California San Francisco (UCSF).

The first part of this consent form gives you a summary of this study. We will give you more details about the study later in this form. The study team will also explain the study to you and answer any questions you have.

**Purpose of the study:** The purpose of this study is to identify effective ways to improve quality of care which includes addressing provider stress and burnout as well as other factors that affect provider interactions with patients such as unconscious bias. This consent form gives you information about this study. The study staff will also explain the study to you and answer any questions. If you agree to participate, we will ask you to sign this consent form. You will get a copy of this form to keep.

**Study Procedures:** If you choose to be in this study, you will first be invited to participate in a baseline survey. After that, you will be assigned to one of two groups based on the group your facility is randomized to. If you are in the intervention group, you will be invited to take part in the intervention activities that includes a two-day training and activities to provide you with peer support and mentorship. These activities will be spread over 6-months and will occur during a convenient time at your health facility. After that we will ask you to participate in 2 or 3 interviews to assess the intervention and how it has affected you. If you are assigned to the control group, you will first be invited to complete 3 interviews after which you will have the opportunity to participate in the intervention activities if you would like to. The data collection will take place in a private space at a time and place that is convenient for you. Here are some more details of the different data collection procedures.

1. **Baseline, midline, and endline survey**: The surveys may ask you questions about your job, the things that make you satisfied or dissatisfied with your job, how you treat pregnant and childbearing women, what causes you to sometimes treat people differently, what stresses you and how you deal with it and what you think can be done to improve your work experience. We may also ask you some questions about yourself and the facility you work in and what can be done to improve your work experience. After the intervention, we will also ask you what you think about the intervention and how we can improve it. The surveys will take place at baseline, midline (6-months after the intervention) and at endline (12-months after the intervention), in a private space and last up to 1 hr. Your responses will be entered on a tablet.
2. **In-depth interviews:** We may follow up to invite you to in-depth interviews (IDIs) during baseline, midline or endline, based on your survey completion. During the IDIs we will ask you more detailed questions and audio record those interviews to make sure we capture all the information. We will then transcribe the recordings and delete the tapes. We will not use the recordings directly so do not worry about someone recognizing your voice. These interviews will take up to 1 hr.
3. **Participation in CPIPE intervention*.*** Study facilities will be assigned to one of two groups in the study- intervention and control. If your facility is assigned to the intervention group, you will participate in the intervention activities after the baseline survey. If you are assigned to the control group, you will have the option to participate in the intervention later after you have completed all the interviews. The intervention includes a training that will last about 2 days followed by peer support and mentorship activities spread over 6-months, which will be conducted at your facility, with the support of your facility head. During the training, you will learn about strategies to manage stress and unconscious bias. In addition, we will conduct a short pre and post training evaluation to help us improve future training sessions.
4. **Future contact, WhatsApp messages and SMS:** As part of the intervention, we will share various resources with you via WhatsApp to reinforce what you learnt during the training. This information will be shared weekly with you initially and less frequently thereafter. You may also be contacted by study staff during the study via phone, WhatsApp, SMS, or in-person visits to remind you of intervention activities or to schedule interviews. We would also like to ask for your permission to be contacted for future studies/activities related to this research. We will maintain your confidentiality and privacy.

**Possible Risks:** There are risks to taking part in a research study. Some of the most likely risks of participation in this study include:

- We expect that you may be uncomfortable participating in some activities or answering some questions in the survey. If you are uncomfortable about a particular activity, you have the right to decline to participate in it.
- There is also possible loss of confidentiality if data is inadvertently shared outside the researchers. But we will take all precautions to make sure this does not happen.

We will tell you more about these risks and other risks of taking part in the study later in this consent form. There may also be risks that we do not know about.

**Possible Benefits**: There may be no benefit to you for participating but we may learn about ways to support providers and patients in Kenya and Ghana. If you participate in the intervention, the intervention might benefit you through helping you to better manage your stress and improving your work experience. We expect this research might lead to better understanding of the types of interventions that can be implemented to improve the experience of health care workers as well as the experience of their clients and lead to overall improvements in quality of health care.

**Your Other Options:** You do not have to participate in this study. If you choose not to participate in this voluntary study, your position or job will not be impacted.

Following is a more complete description of this study. Please read this description carefully. You can ask any questions you want to help you decide whether to join the study. If you join this study, we will give you a signed copy of this form to keep for future reference.

**DETAILED STUDY INFORMATION**

This part of the consent form gives you more detailed information about what the study involves.

**What are some things you should know about your participation and research?**

Before you take part in this study, there are some things I want you to know. You do not have to be in this study if you do not want to and you may stop being in the study at any time. You can also opt out of any part of the study, at any time; no one will be upset with you. Participation in this study will not affect your position in any way. Data collected at previous visits will continue to be used for the study; this data will not have information that can be easily used to identify you. Also, the study researchers may stop you from taking part in this study at any time if they believe it is in your best interest, if you do not follow the study rules, or if the study is stopped.

**What is the purpose of this study?**

The purpose of this research is to identify effective ways to improve quality of care that includes addressing provider concerns such as stress and burnout as well as other factors that affect provider interactions with patients such as unconscious bias. You are being asked to participate because you are a provider who may be impacted by stress and unconscious bias and could provide useful insights to identify the impact of the study intervention.

**Are there any reasons you should not be in this study?**

You should not be in this study if you have been working as a healthcare provider for less than six months and/or if you plan to change jobs within the next six months.

**How long will you participate in this study and with how many other participants?**

Your participation in this study will last for about 12 months. This time may also be shorter. There will also be approximately 400 providers, across Kenya and Ghana, participating in the study with you.

**What if we learn about new findings or information during the study?**

You will be given any new information gained during the study that might affect your willingness to continue your participation.

**Confidentiality**

The information collected will be kept confidential and used only for the purposes of this study. Personal information like your name and phone number will only be used to contact you about the study and will not be linked directly to data we collect during surveys or interviews. We will only link data collected from you at multiple times using an ID number to protect your identity. Only the research study staff will have access to the information. At the end of the study, your personal information will be deleted such that there will be no way to directly link your name with your data.

**How will my information be used?**

Researchers will use your information to conduct this study. Once the study is done using your information, we may use the information collected for future research studies or share them with other researchers so they can use them for other studies in the future. We will not share your name or any other personal information. We cannot guarantee that this will prevent future researchers from determining who you are. We will not ask you for additional permission to share this de-identified information.

**How will information about you be protected?**

The information you give us will be private to the extent allowed by the law. We will not write your name in any of our reports and will use a number in place of your names. All of your information will either be stored in locked cabinets or on secure computers, accessed only by authorized study staff. Any audio recordings of interviews or discussions for the qualitative study will be kept only until they are transcribed, then the recordings will be destroyed. The transcriptions will not include your name or any information that could identify you, but only your study ID number. Screening, enrollment, and follow-up events will take place at facilities in the study area. The study will not maintain a permanent location, apart from the offices. You will meet only with the designated study staff, and all interviews will be conducted in a private area to ensure privacy. Your study identification number will be the only form of identification used. A description of this trial will be available on http://www.ClinicalTrials.gov, as required by U.S. Law. This website will not include information that can identify you.

**What will happen if you are injured by this research?**

All research involves a chance that something bad might happen to you. But we expect this risk to be really minimal in this study. If you think you have been harmed or injured by the study, please contact the study hotline number on the first page.

**Will I be paid for taking part in this study?**

You will not be paid for taking part in this study. But you will receive Ksh. 600 as appreciation for your time each time you participate in the baseline, midline and endline surveys and if you participate in an interview. If you participate in the study intervention, you will receive a transport reimbursement of about Ksh 1000 for each of the training days you participate in. You will also receive food and refreshments during training. During study activities in your facility, we may also provide refreshments. We will at no point ask you for any money to be in this study.

**Who is sponsoring this study?**

This research is funded by the United States National Institutes of Health.

**What if you have questions about this study?**

Should you have questions or concerns about the study, please call us at the study hotline number at +254 790300073

**What if you have questions about your rights as a research participant?**

If you wish to ask questions about your rights as a participant to someone other than the staff members may contact the KEMRI Scientific and Ethics Review Unit at +254-0202 722 541 or +254-0717 719 477 or [seru@kemri.org](mailto:seru@kemri.org).

**Participant’s Agreement:**

I have read the information provided above and asked all the questions I have at this time; these questions are answered.

I voluntarily agree to participate in this research study. I give my permission to participate in the following activities, knowing that I can decide to withdraw my consent for **any or all of them** at any time:

| ***Required for participation in the study:*** | Participant: Write initials | |
| --- | --- | --- |
| Participation in the baseline, midline, and endline surveys (**for provider cohort)** | **Yes** | **No** |
| Participation in the CPIPE training and refresher activities (if assigned to **intervention group)** | **_____**  **Yes** | **____**  **No** |
| **Participant: If provider cohort and NO to any of the above, do not sign form!!!** | | |
| ***Not required for participation in the study:*** |  | |
| Participate in audio recorded in-depth interviews | **_____**  **Yes** | **_____**  **No** |
| To be contacted for future study activities | **_____**  **Yes** | **_____**  **No** |

Signatures:

| **STUDY STAFF** |
| --- |
| Name of Study Staff (printed) Position  Signature of Study Staff Date |

| **PARTICIPANT** |
| --- |
| Name of Research Participant (printed)  Signature of Research Participant Date |

| **WITNESS** |
| --- |
| If the participant is unable to read and/or write, an impartial witness must be present during the screening consent discussion. After the written informed consent form is read and explained to the participant, and after he or she has orally consented to his or her participation in this screening and has either signed the consent form or provided his or her fingerprint, the witness must sign and personally date the consent form. By signing the consent form, the witness attests that the information in the consent form and any other written information was accurately explained to, and apparently understood by, the participant, and that consent was freely given.  Name of Witness (printed)  Signature of Witness Date |

**For study team:** Please select the cohort type:

| *Select only one* | *Select only one* |
| --- | --- |
| Ο Intervention | Ο Provider |
| Ο Control | Ο Facility leader |
